# Effect and Mechanism of Catalpol on Remyelination via Regulation of the NOTCH1 Signaling Pathway

**DOI:** 10.3389/fphar.2021.628209

**Published:** 2021-02-23

**Authors:** Yaqin Sun, Jing Ji, Zheng Zha, Hui Zhao, Bing Xue, Liangyun Jin, Lei Wang

**Affiliations:** ^1^ School of Traditional Chinese Medicine, Beijing Key Lab of TCM Collateral Disease Theory Research, Capital Medical University, Beijing, China; ^2^ Core Facility Center, Capital Medical University, Beijing, China

**Keywords:** catalpol, multiple sclerosis, remyelination, oligodendrocytes, cuprizone, Notch1 signaling pathway

## Abstract

Promoting the differentiation of oligodendrocyte precursor cells (OPCs) is important for fostering remyelination in multiple sclerosis. Catalpol has the potential to promote remyelination and exert neuroprotective effects, but its specific mechanism is still unclear. Recent studies have shown that the NOTCH1 signaling pathway is involved in mediating OPC proliferation and differentiation. In this study, we elucidated that catalpol promoted OPC differentiation *in vivo* and vitro and explored the regulatory role of catalpol in specific biomolecular processes. Following catalpol administration, better and faster recovery of body weight and motor balance was observed in mice with cuprizone (CPZ)-induced demyelination. Luxol fast blue staining (LFB) and transmission electron microscopy (TEM) showed that catalpol increased the myelinated area and improved myelin ultrastructure in the corpus callosum in demyelinated mice. In addition, catalpol enhanced the expression of CNPase and MBP, indicating that it increased OPC differentiation. Additionally, catalpol downregulated the expression of NOTCH1 signaling pathway-related molecules, such as JAGGED1, NOTCH1, NICD1, RBPJ, HES5, and HES1. We further demonstrated that *in vitro*, catalpol enhanced the differentiation of OPCs into OLs and inhibited NOTCH1 signaling pathway activity. Our data suggested that catalpol may promote OPC differentiation and remyelination through modulation of the NOTCH1 pathway. This study provides new insight into the mechanism of action of catalpol in the treatment of multiple sclerosis.

## Introduction

Multiple sclerosis (MS), a demyelinating disease, affects the central nervous system (CNS), especially the white matter, and is characterized by immune cell infiltration, demyelination, oligodendrocyte (OL) loss and axonal destruction (McQualter and Bernard, 2007; Jurasic et al., 2019). Demyelination caused by immune inflammation results in severe neurological dysfunction in patients with MS (Stanojlovic et al., 2016). Promoting remyelination is important for the restoration of neurological function in MS. During the development of MS, lesions contain enough oligodendrocyte precursor cells (OPCs), which differentiate into myelinating OLs, to form a new myelin sheath around the injured axon. However, this process fails due to interference by various factors. A previous study revealed that activation of the NOTCH1 signaling pathway is one of these factors. The NOTCH1 signaling pathway affects the development and progression of the disease in the CNS by regulating the proliferation, differentiation and apoptosis of stem cells. NOTCH1 binds to the ligand JAGGED1to activate downstream signaling pathways and then directly converts extracellular information into changes in nuclear gene expression (Zhang et al., 2009; Paganin and Ferrando, 2011), which may hinder OPC differentiation and remyelination.

Doctors often use glucocorticoids and plasma exchange to alleviate symptoms, shorten the course of the disease, reduce the degree of disability and prevent complications in the acute phase of MS. In the remission phase, teriflunomide, interferon, fingolimod and other disease-modifying therapies (DMTs) are used to control disease progression and reduce recurrence. However, these therapies cannot prevent CNS neurodegeneration (Faissner et al., 2019). Currently, there are no safe and effective strategies to promote remyelination to improve nerve function (Plemel et al., 2017; Katsara and Apostolopoulos, 2018).

Catalpol, also known as catalpinoside, is the main active ingredient of the traditional Chinese herbal medicine *Rehmannia glutinosa* (Gaertn.) DC*.*. Catalpol has anti-inflammatory and antioxidant properties and exerts neuroprotective effects, improving neurocognitive function (Xia et al., 2017). Administration of 10 mg/kg or 20 mg/kg catalpol for 14 days produces significant antidepressant effects in a mouse model of depression through the serotonin pathway (Wang et al., 2014). The antidepressant effects of catalpol may be related to repair of the hypothalamic-pituitary-adrenal (HPA) axis and increased expression of brain-derived neurotrophic factor (BDNF) (Wang et al., 2015). Catalpol can also protect forebrain neurons from neurodegeneration and enhance memory by increasing BDNF expression (Liu et al., 2006; Wang et al., 2009; Wan et al., 2013). In an experimental model of Parkinson’s disease, catalpol increases the concentration of striatal dopamine and the level of glial cell-derived neurotrophic factor (GDNF) (Xu et al., 2010), thereby exerting its neuroprotective functions.

We have found that catalpol can promote remyelination in experimental autoimmune encephalomyelitis (EAE) mice by upregulating the expression of the transcription factors OLIG1 and OLIG2, as well as increasing the proliferation, migration and differentiation of OPCs *in vitro* (Yuan et al., 2015; Yang et al., 2017). However, the mechanism is unclear. Therefore, in this study, we will use a mouse model of demyelination induced by cuprizone (CPZ) and OPCs *in vitro* to explore whether catalpol promotes remyelination by regulating the NOTCH1 signaling pathway.

## Materials and Methods

### Animals

Female specific pathogen-free (SPF) C57BL/6J mice (aged 6–8 weeks) were provided by Beijing Weitong Lihua Experimental Animal Technology Co., Ltd. [SCXK (Beijing) 2016-0006]. The mice were housed in an SPF laboratory at the Experimental Animal Center of Capital Medical University [SYXK (Beijing) 2018-0003] at a stable temperature and humidity and provided solid rodent food and water. Animal experiments were approved by the Animal Experiments and Experimental Animal Welfare Committee of Capital Medical University (AEEI-2015-185).

### Drugs

Special feed containing 0.2% CPZ (Sigma, United States) produced by Beijing Keao Xieli Feed Co., Ltd. [Beijing Feed Certification (2014) 06054] was used for this experiment after disinfection by cobalt-60 irradiation. For *in vivo* studies, catalpol was purchased from Nanjing Dilge Pharmaceutical Technology Co., Ltd. and used in doses of 20 mg/kg, 40 mg/kg and 80 mg/kg, and its purity was ≥85%. For *in vitro* studies, the standard substance of catalpol was provided by Target Molecule Corp (T2780) and used in the concentrations of 0–100 μM, and its purity was ≥ 99%.

### Establishment and Treatment of the CPZ-Induced Demyelination Mouse Model

**FIGURE 1:**
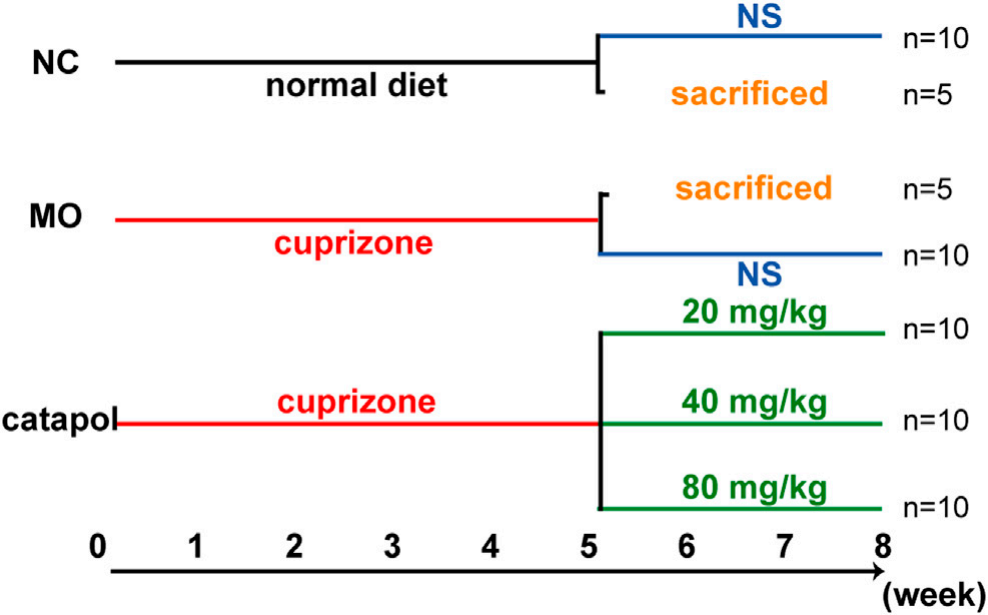
The design scheme of experimental protocol. At the end of the fifth week, five mice in the NC group and MO group were randomly selected and sacrificed; The remaining mice continued to be raised. Black represents a normal diet, red represents a diet containing 0.2% CPZ, blue means a normal diet plus gavage of normal saline, and green represents a normal diet plus gavage of catalpol.

Sixty mice were randomly divided into five groups: the normal control (NC) group (n = 15), the model (MO) group (n = 15), and the 20 mg/kg, 40 mg/kg and 80 mg/kg catalpol groups (n = 10/group). The mice in the NC group were given normal feed, and the mice in the other five groups were given feed containing 0.2% CPZ. After 5 weeks, the levels of relevant indicators were assessed in five mice from each of the NC group and the MO group. From the 6^th^ week, the remaining mice were provided normal feed. The catalpol groups were administered the corresponding dose of catalpol daily by gavage, whereas the NC and MO groups were given the same volume of normal saline. Biological materials were collected after 8 weeks (Figure 1).

### Body Weight Measurement and the Rotarod Test

From the start of modeling, the body weight of each mouse was measured twice a week. For three days before modeling, the mice were subjected to balance training on a rotating device twice a day. After the mice had been adapted to the device, their motor abilities were tested twice a week. The rotation speed was slowly increased from 5 rpm to 40 rpm over 3 min. When a mouse fell off or held onto the rotating rod and for two or more rotations, the experiment was ended. The time spent on the rod by the mice was recorded to monitor changes in coordination and balance (Fan et al., 2018).

### Luxol Fast Blue Staining

The mice were anesthetized with 4% chloral hydrate and perfused with 40 g/L paraformaldehyde for 30 min, and their tissues were embedded with paraffin. Five-micron -thick coronal slices of the brain located in the corpus callosum were prepared. After being washed with phosphate-buffered saline (PBS), the brain slices were baked at 60°C for 2 h, placed in 1:1 ethanol and chloroform for 4 h, and then transferred to 95% ethanol. After dehydration, the sections were placed in LFB solution overnight, rinsed, and then subjected to color development with 0.05% lithium carbonate and 70% alcohol. After being rinsed, the sections were counterstained with cresyl violet for 5 min, rinsed with water, dehydrated, removed, and sealed for observation.

### Primary OPC Culture

Newborn Sprague-Dawley (SD) rats were provided by Beijing Weitong Lihua Experimental Animal Technology Co., Ltd. [SCXK (Beijing) 2016-0006]. OPCs were isolated and purified by a method involving B104-conditioned medium. Mixed glial cells were isolated from the cortices of newborn rats. After being cultured in Dulbecco's modified Eagle medium (DMEM) containing 10% fetal bovine serum (FBS) for 3 days, the mixed glial cells were cultured with modified OPC growth medium (mOGM) containing 15% B104-conditioned medium. The OPCs were isolated and purified by a chemical-based separation procedure when they had proliferated enough.

### Cell Counting Kit (CCK)-8 Assay

One hundred microliters of a single-cell suspension containing OPCs (1.5 × 10^4^) was seeded in PPL-coated 96-well plates for 24 h and then treated with catalpol (0, 1, 2.5, 5, 10, 20, 40, 80, or 100 μM) for 24 h, 48 h or 72 h. Then, cell viability was assessed by a CCK-8 kit according to the manufacturer’s instructions. The optical density (OD) values at 450 nm were measured with a microplate reader and normalized to those of the control groups.

### Transmission Electron Microscopy

After perfusion, the corpus callosum tissues of the mice were separated on ice, cut into approximately 1 × 1 × 3 mm^3^ pieces, placed in 2.5% glutaraldehyde for 2 h, and then rinsed with 0.1 M PB three times. Then the corpus callosum tissues were fixed with 1% osmium acid, dehydrated in alcohol, soaked for 20 min, embedded in embedding agent, subjected to melt impregnation and treated with pure embedding agent. The embedded samples were sliced with an ultrathin microtome and then stained and coverslipped. The ultrastructure of the myelin sheath was observed with an electron microscope (JEM-2100, JEOL, Tokyo, Japan). We randomly selected at least 80 axons from each group, used professional image analysis software (Image-Pro Plus, IPP) to measure the diameter of the myelin sheath and axon in each field of view, and calculated the G-ratio (axon/axon diameter + myelin sheath diameter) to evaluate demyelination.

### Immunofluorescence

The mouse brain slices were dewaxed, hydrated, incubated in citric acid for 20 min for antigen repair, cooled to room temperature (RT), and blocked with 10% goat serum at 37°C for 60 min. Goat anti-OLIG2 (1:100; R&D, MN, United States), mouse anti-CNPase (1:200, Abcam, Cambridge, United Kingdom), rabbit anti-MBP (1: 200, Abcam, Cambridge, United Kingdom), and rabbit anti-GFAP (1:400, Abcam, Cambridge, United Kingdom) primary antibodies were added dropwise, and the slices were incubated at 4°C for 48 h. After the slices were rewarmed at 37°C for 60 min, they were incubated with corresponding fluorescently labeled IgG antibodies (Alexa Fluor 488-conjugated donkey anti-rabbit or mouse IgG [1: 200], Cy3-labeled donkey anti-goat IgG [1: 200], or Alexa Fluor 488-conjugated goat anti-rabbit IgG [1: 200]). Then, the slices were sealed with DAPI solution. Representative images were obtained with the Pannoramic SCAN digital slice scanner and analysis software (3DHISTECH, Hungary), and ImageJ was used to determine the expression of the abovementioned proteins in the selected area for further data analysis.

A total of 500 μl of a single-cell suspension containing OPCs (4.5 × 10^4^) was plated in 24-well plates with coated glass coverslips and incubated for 12 h. Then, catalpol (0, 1, 2.5, 5, 10, 20, 40, 80, or 100 μM) was added. After 24 h, 48 h or 72 , the OPCs on coverslips were fixed with 4% paraformaldehyde for 30 min at RT and treated with 0.5% Triton for 10 min. After being rinsed with PBS, the coverslips were blocked with 5% bovine serum albumin (BSA) for 1 h and then incubated with a rabbit anti-MBP antibody (1:200, Abcam, Cambridge, United Kingdom) at 4°C overnight. Then, the cells were washed with PBS and incubated with Alexa Fluor 488-conjugated donkey anti-rabbit IgG (1:200) at RT for 1 h. The cell nuclei were stained with DAPI for 5 min. Immunoreactivity was observed using a fluorescence microscope. All images were analyzed with ImageJ software.

### Western Blot Analysis

OPCs (9 × 10^5^ cells/well in 6-well plates, treated with 40 μM catalpol according to the method used for immunofluorescence) and mouse brain tissues (containing the corpus callosum, cortex and hippocampus, 30–50 µg) were lysed with RIPA lysis buffer. The protein concentrations were measured using the Bicinchoninic Acid (BCA) Protein Assay Kit and normalized. The proteins were separated by SDS-PAGE and transferred onto PVDF membranes. After being blocked with 5% skim milk for 1 h, the membranes were incubated with the following primary antibodies overnight at 4°C: rabbit anti-JAGGED1 (1:1,000, CST, 2620), rat anti-NOTCH1 (1:1,000, CST, MA, United States), rabbit anti-NICD1 (1:1,000, CST, MA, United States), rabbit anti-RBPJ (1:1,000, CST, MA, United States), rabbit anti-HES1 (1:1,000, CST, MA, United States), rabbit anti-HES5 (1:1,000, CST, MA, United States), mouse anti-ACTB (1:20,000, Gene Tex, CA, United States), and mouse anti-TUBB (1:20,000, Proteintech, Chicago, United States). After being washed, the membranes were incubated with corresponding IgG antibodies (1:10,000) at RT for 1 h. The proteins were exposed by a gel chemiluminescence imaging analysis system using enhanced chemiluminescence (ECL) reagent. ImageJ software was used to process the images, determine the grayscale values of the bands, and semiquantitatively analyze the relative expression of each protein.

### Quantitative RT-PCR

Total RNA was extracted from 50 μg samples with the One-Step qRT-PCR kit (Toyobo, Osaka, Japan), and then the concentration was measured. RT-PCR mixtures were prepared, and qRT-PCR was performed by the CFX96 TM Real-Time PCR instrument to confirm the mRNA expression levels of the target genes. *Actb* was used as an internal reference for RNA. mRNA expression levels were quantified using the Bio-Rad CFX Real-Time system and analyzed using CFX management software v2.0 (Bio-Rad, Hercules, CA) and the 2^-△△^Ct method.

The sequences of the primers used in this study were as follows: *Notch1*: FWD-GTCCCCTGGGTTTCTCTG and REV-GCAGCGGCACTTGTACTC;*Jag1*: FWD- GACCGTAATCGCATCGTAC  andREV-CCTGAGTGAGAAGCCTTTTC;*RBPJ*: FWD-AAGCGGATAAAGGTCATCTC and REV-AAATGCTCCCCACTGTTG; *Hes1*:FWD-AAGCTAGAGAAGGCAGACATTC and REV-GTAGGTCATGGCGTTGATC; *Hes5*: FWD-GGTACAGTTCCTGACCCTGC and REV-AGCAGCAGCAGCCTTAGC; and *Actb:* FWD-TGCGTGACATCAAAGAGAAG and REV-AGAAGGAAGGCTGGAAAAG.

### Statistical Analysis

All data are presented as the mean ± SEM. Statistical analyses were performed by one-way analysis of variance and Tukey’s HSD multiple comparison test using GraphPad Prism 7.0 software. For all statistical tests, *p-*values < 0.05 were considered statistically significant.

## Results

### Successful Establishment of the CPZ-Induced Demyelination Model

**FIGURE 2:**
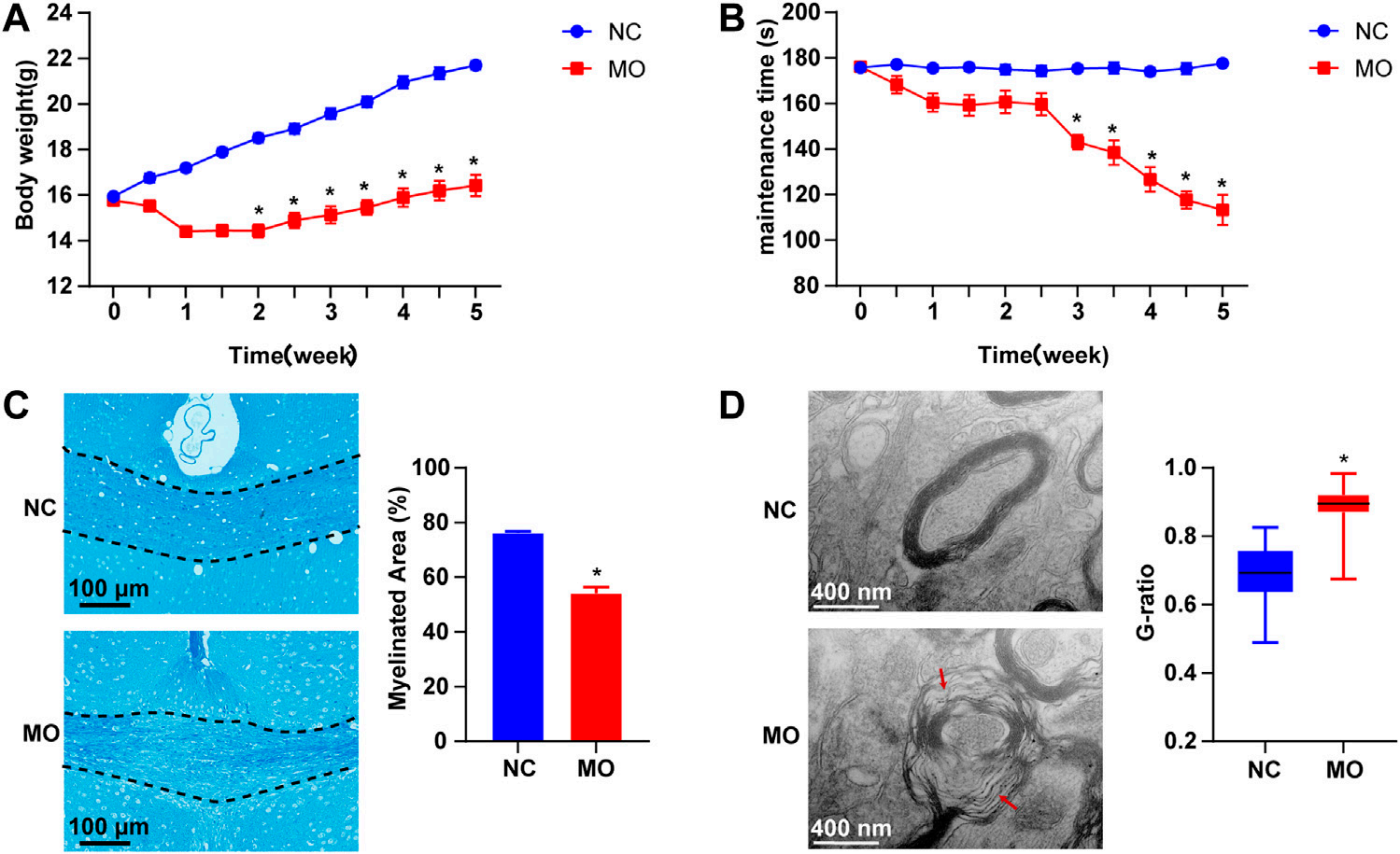
The model of CPZ-induced demyelination. **(A)** Changes in body weight (n = 15/each group), **(B)** rotating rod experiment in mice (n = 13/each group), **(C)** changes in demyelination of the corpus callosum in mice: FLB × 200 times and statistical results of myelinated area (n = 3/each group), **(D)** changes in myelin ultrastructure (TEM×12,000 times) and myelin sheath G-ratio (n = 3, 80 myelin sheaths randomly selected from each group). The data are expressed as mean ± SEM, compared with the NC group, *p < 0.05.

The body weights of the mice in the NC group increased over time. The weights of the mice in the MO (5 weeks) group gradually decreased to the lowest by the end of the second week and then slowly increased. There was a statistically significant difference in body weight between the NC and MO (5 weeks) groups (*p* < 0.05, Figure 2A). The results of the rotarod test showed that the time spent on the rod by the mice in the NC group remained basically the same over time. However, from the 3rd week, the time spent on the rod by the mice in the MO (5 weeks) group decreased significantly (*p* < 0.05, Figure 2B).

LFB staining indicated that in the mice in the NC group, the myelin sheaths were tight and properly arranged and that there was a large amount of staining; however, in the MO (5 weeks) group, myelin staining in the corpus callosum was notably reduced and sparse, or even absent (*p* < 0.05, Figure 2C). According to the results of TEM, in the NC group, the myelin sheaths were clear and dense and no demyelination or axonal atrophy was observed, whereas loose myelin sheaths with lamellar separation, a decreased ring density, axonal atrophy and significantly higher G-ratios were observed in the MO (5 weeks) group (Figure 2D, *p* < 0.05). Based on the above indicators, the mouse model of CPZ-induced demyelination was successfully established.

### Catalpol Increased the Body Weights and Improved the Motor Functions of Demyelinated Mice

**FIGURE 3:**
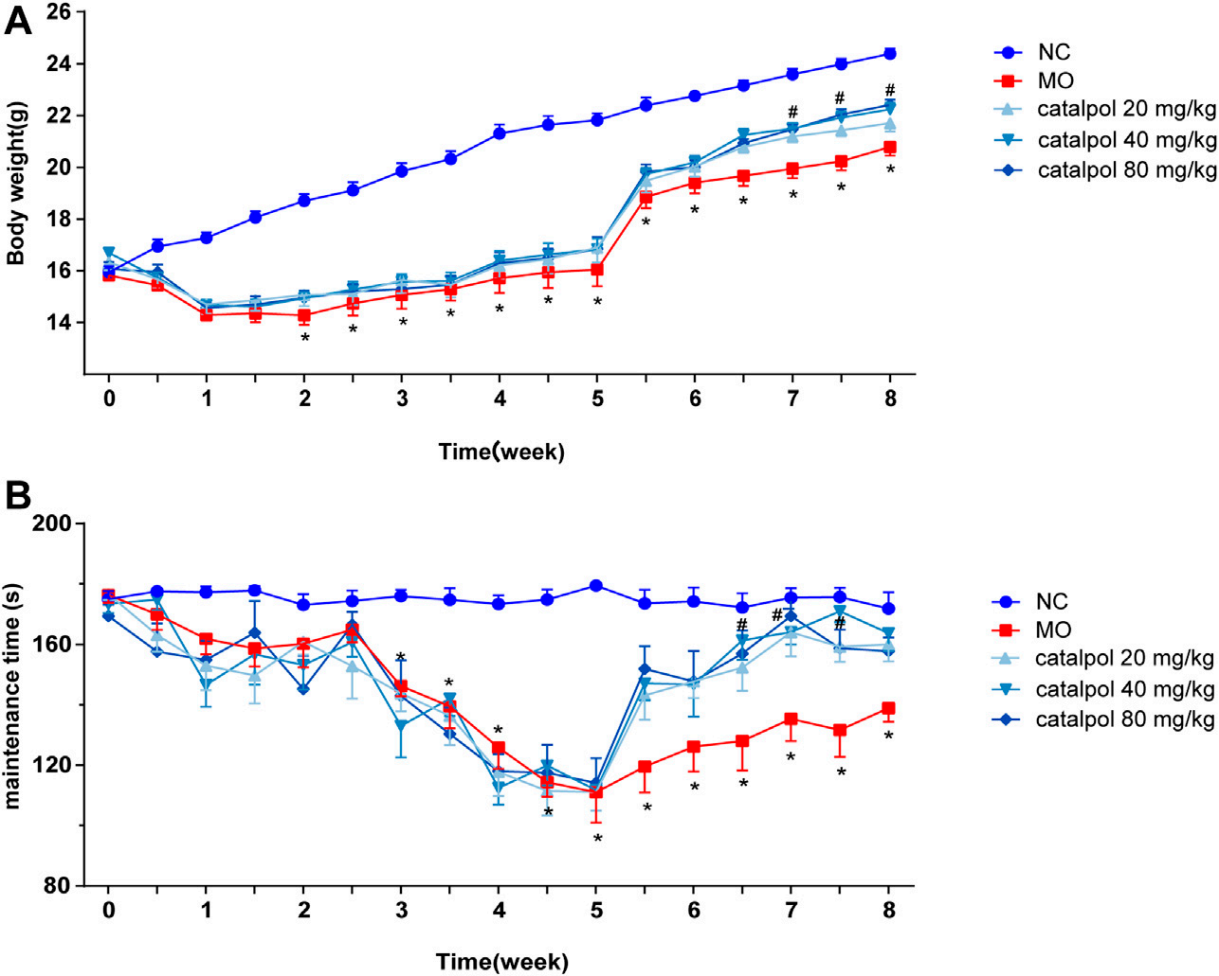
Different concentrations of catalpol improved the body weight and exercise ability of CPZ-induced demyelinated mice. **(A)** Changes in body weight of mice in each group (n = 10), **(B)** changes in the time of turning rods of mice in each group (n = 8). The data are expressed as mean ± SEM, compared with the NC group, *p < 0.05; compared with MO group, ^#^ *p* < 0.05.

After the mice were given normal feed beginning in the 6^th^ week, the body weights of the mice in the NC group continued to increased, and those of the mice in the MO group recovered slightly (*p* < 0.05). The body weights of the mice in the 40 mg/kg and 80 mg/kg catalpol groups recovered quickly. From the 7^th^ week, there were significant differences in body weight between the catalpol groups and the MO group (*p* < 0.05, Figure 3A). The rotarod test results showed from the 6^th^ week, the time spent on the rod by the mice in the NC group remained relatively stable, whereas that spent by the mice in the MO group improved but was still significantly shorter than that spent by the mice in the NC group (*p* < 0.05). The time spent on the rod by the mice in the 20 mg/kg catalpol group was significantly longer than that spent by the mice in the MO group from the 7^th^ week (*p* < 0.05), while the time spent on the rod by the mice in the 40 mg/kg and 80 mg/kg catalpol groups was significantly longer than that spent by the mice in the MO group beginning at six and a half weeks (*p* < 0.05, Figure 3B).

### The Effect of Different Concentrations of Catalpol in Reducing Myelination in CPZ-Induced Demyelination Mice

**FIGURE 4:**
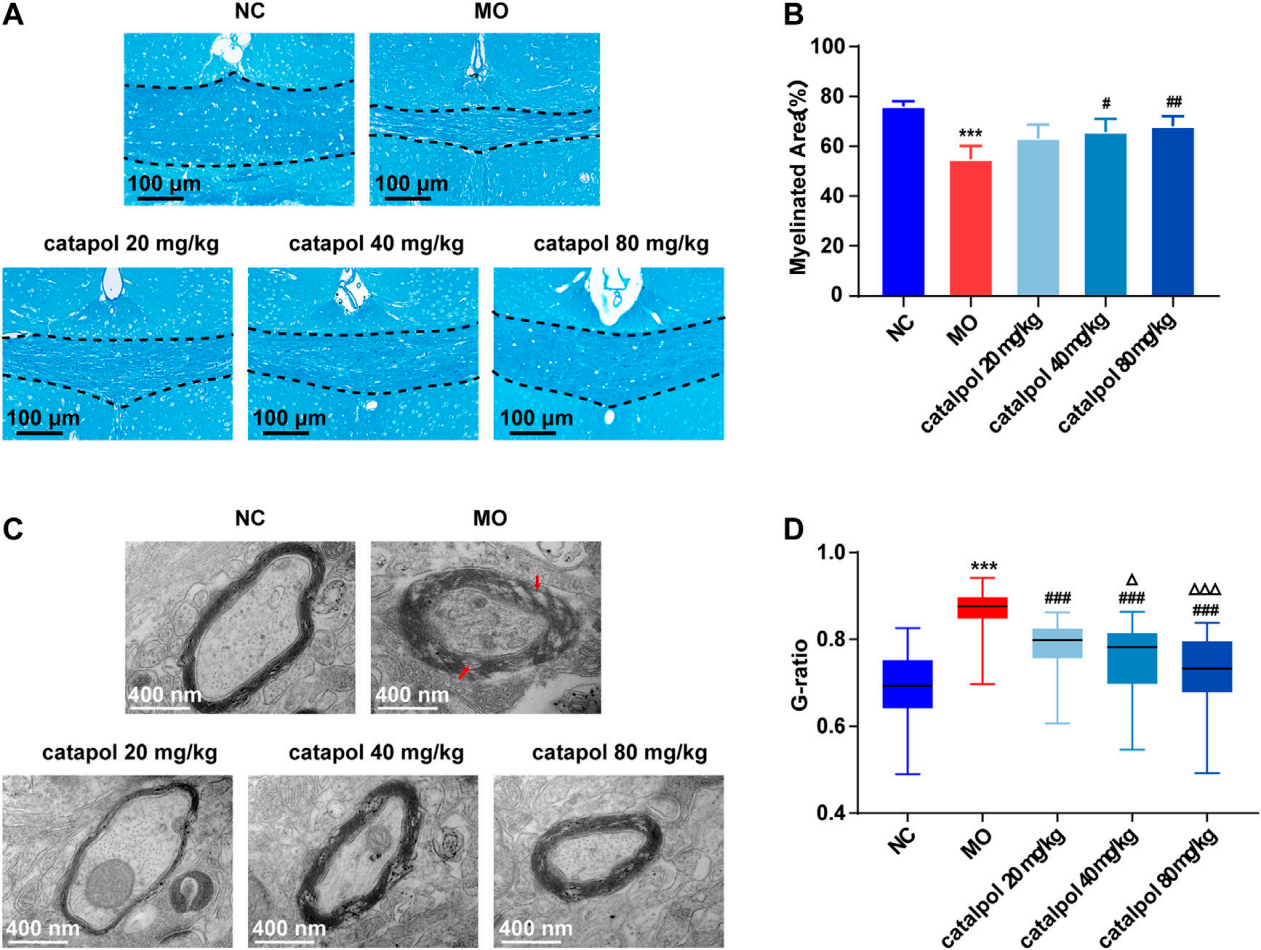
Catalpol at different concentrations significantly promoted myelination in demyelinated mice. **(A)** Changes in demyelination of the corpus callosum in mice (FLB × 200 times,n = 3/each group), **(B)** statistical results of myelinated area of the corpus callosum in each group (n = 3), **(C)** changes in the ultrastructure of myelin sheath (TEM×12,000 times, n = 3/each group), **(D)** changes in G-ratio of myelin sheath in each group (n = 3, randomly selecting 80 myelin sheaths each group for statistics). The data are expressed as mean ± SEM. Compared with NC group, ***p < 0.001; compared with MO group, ^#^ *p* < 0.05, ^##^ *p* < 0.01, ^###^ *p* < 0.001; compared with 20 mg/kg catalpol group, ^△^ *p* < 0.05, ^△△△^ *p* < 0.001.

At the 8^th^ week, partial remyelination was observed in the corpus callosum in mice in the MO group. The loss of LFB staining of myelin and myelin lamination was slightly delayed in the MO group, but a significant difference compared with the NC group (*p* < 0.001) was still observed. The stained myelin sheaths were significantly denser and better arranged (Figure 4A), the demyelinated area was significantly smaller (Figure 4B) in the catalpol administration groups than in the MO group (*p* < 0.05). The three catalpol groups exhibited loosening of the lamellar structure of myelin to varying degrees and decreases in ring density. However, these changes were less severe in the three catalpol groups than in the MO group (Figure 4C). The G-ratios were significantly lower in the catalpol groups than in the MO group (*p* < 0.05, *p* < 0.001), with the 40 mg/kg and 80 mg/kg catalpol groups exhibiting more significant changes (*p* < 0.05, *p* < 0.001, Figure 4D).

### Catalpol Upregulated the Expression of CNPase and MBP in the Corpus Callosum in Demyelinated Mice

**FIGURE 5:**
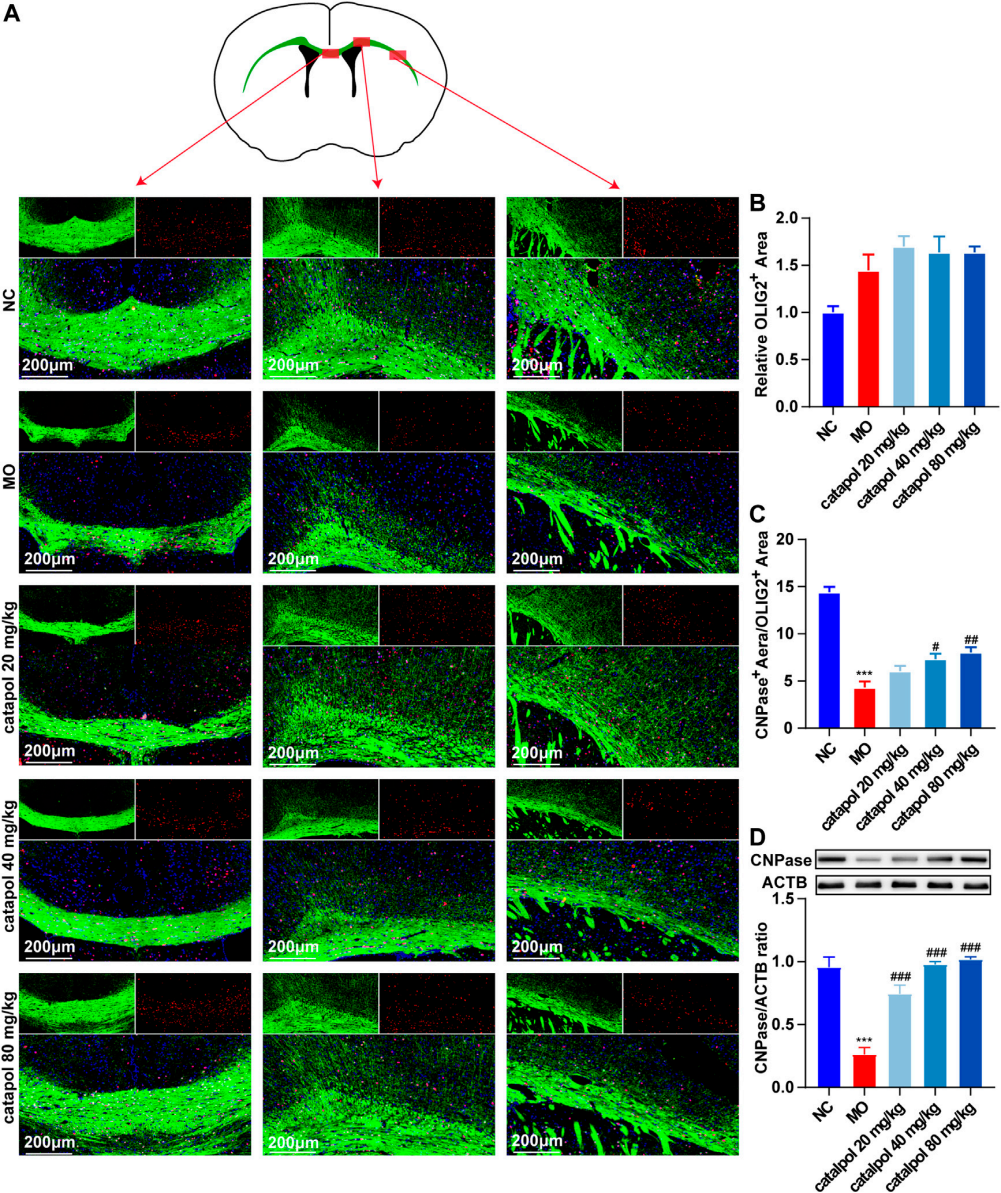
Catalpol at different concentrations up-regulated CNPase in the corpus callosum of demyelinated mice. **(A)** Immunofluorescence localization of CNPase and OLIG2 in the brain. CNPase (green) and OLIG2 (red) were labeled with fluorescent secondary antibodies, and the nuclei were labeled with DAPI. **(B)** Quantitative analysis of the fluorescent expression of OLIG2, **(C)** quantitative analysis of fluorescent expression of CNPase, **(D)** changes of CNPase protein in the brains. The data are expressed as mean ± SEM (n = 3/each group), compared with NC group, ***p < 0.001; compared with MO group, ^#^ *p* < 0.05, ^##^ *p* < 0.01, ^###^ *p* < 0.001.

**FIGURE 6:**
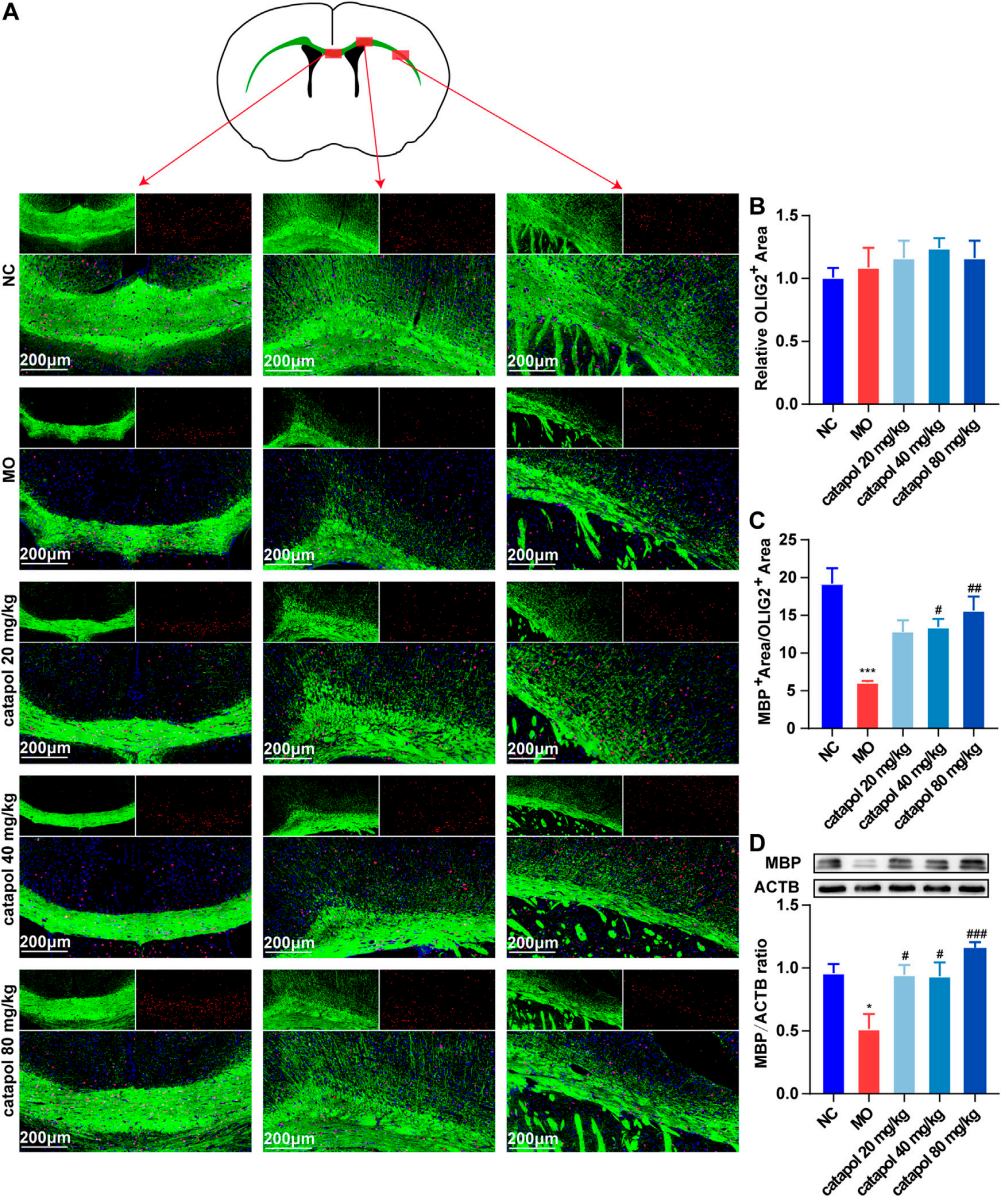
Catalpol at different concentrations up-regulated MBP in the corpus callosum of demyelinated mice. **(A)** Immunofluorescence localization of MBP and OLIG2 in the brain. MBP (green) and OLIG2 (red) were labeled with fluorescent secondary antibodies, and the nuclei were labeled with DAPI. **(B)** Quantitative analysis of the fluorescent expression of OLIG2, **(C)** quantitative analysis of fluorescent expression of MBP, **(D)** changes of CNPase protein in the brains. The data are expressed as mean ± SEM (n = 3/each group), compared with NC group, *p < 0.05, ***p < 0.001; compared with MO group, ^#^ *p* < 0.05, ^##^ *p* < 0.01, ^###^ *p* < 0.001.

CNPase and MBP are markers of OL differentiation and maturity, respectively. OLIG2, which is essential for cell fate choices, is a transcription factor in OLs (Gouvea-Junqueira et al., 2020). The expression levels of CNPase/OLIG2 and MBP/OLIG2 in the brains of mice in each group were detected by immunofluorescence to evaluate the differentiation and maturation of OPCs. The experimental results indicated that there was no difference in the expression of OLIG2 in the corpus callosum between groups (Figure 5B). This finding further indicates that insufficient differentiation of OPCs, not a lack of OPCs, causes the failure of remyelination in MS. Furthermore, the expression levels of CNPase/OLIG2 in the mice in the MO group were decreased compared to those in the NC group (Figure 5A, *p* < 0.001); CNPase/OLIG2 levels in the catalpol treatment groups were increased to a certain extent. Catalpol had more robust effects at 40 mg/kg and 80 m/kg than at 20 mg/kg (*p* < 0.01). The expression of CNPase protein was also measured by WB analysis (Figure 5D), and similar results were obtained. The MBP measurement results were basically consistent with those of CNPase. There was no difference in the expression of OLIG2 between groups (Figure 6B), but there were obviously fewer MBP^+^/OLIG2^+^ cells in the MO group than in the NC group and the catalpol groups (Figure 6A, *p* < 0.001). Analysis of MBP protein expression in the brain further confirmed the effect of catalpol on promoting the differentiation of OPCs (Figure 6D).

### Catalpol Downregulated the Expression of GFAP in the Brains of Demyelinated Mice

**FIGURE 7:**
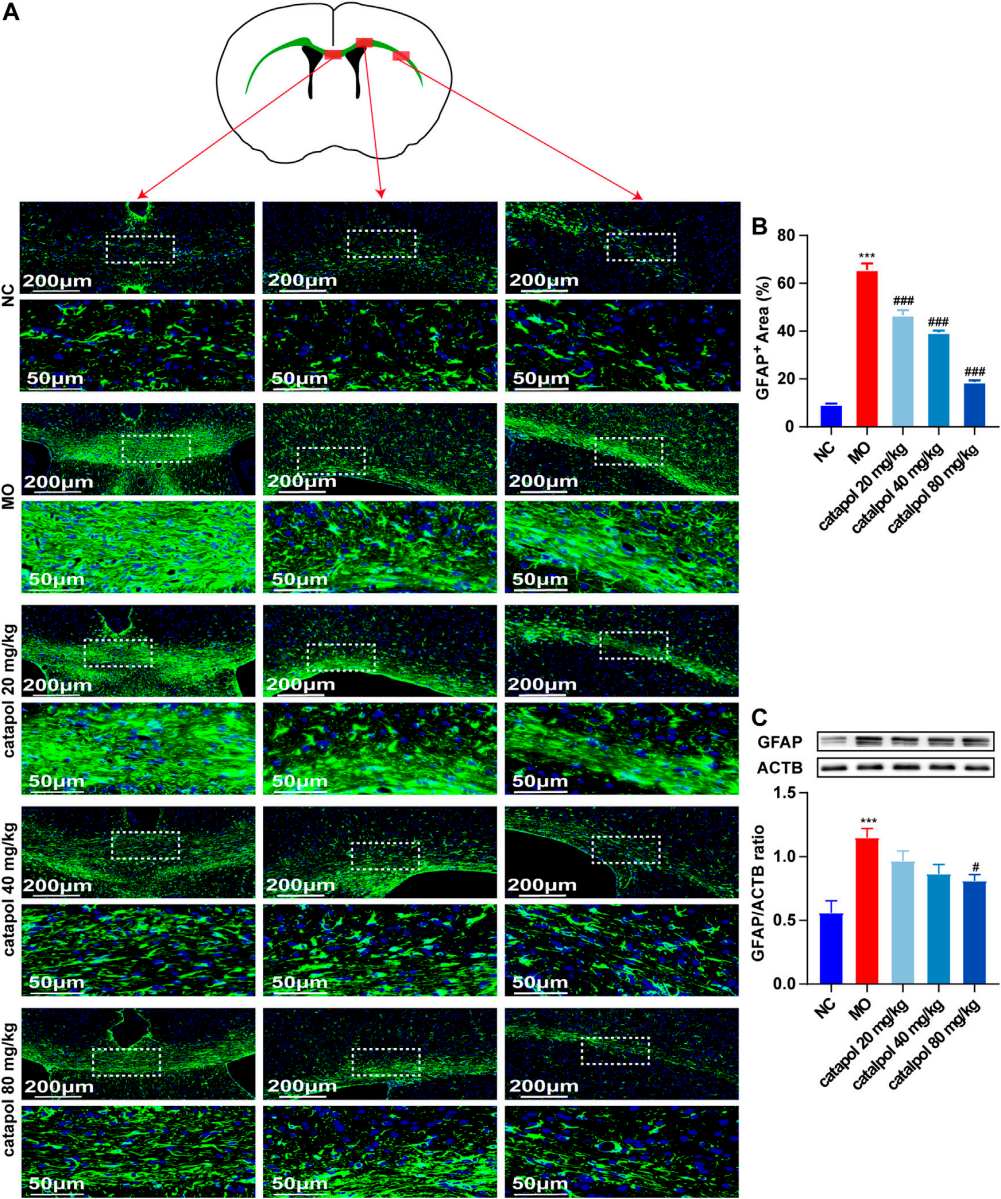
Catalpol at different concentrations down-regulated GFAP in the corpus callosum of demyelinated mice. **(A)** Immunofluorescence localization of GFAP in the brain. GFAP (green) was labeled with fluorescent secondary antibodies, and the nuclei were labeled with DAPI. **(B)** Quantitative analysis of the fluorescent expression of GFAP, **(C)** changes of GFAP protein in the brains. The data are expressed as mean ± SEM (n = 3/each group), compared with NC group, ***p < 0.001; compared with MO group, ^#^ *p* < 0.05, ^###^ *p* < 0.001.

GFAP is a marker of astrocytes. Studies have found that astrocytes participate in the first line of defense against the early stages of immune inflammation in MS (Farina et al., 2007; Brosnan and Raine, 2013; Ponath et al., 2017) and have neuroprotective effects (Colombo and Farina, 2016). However, the activation of astrocytes during the remyelination stage is harmful (Mayo et al., 2012). Reactive astrocytes form an astroglial scar with recruited chondroitin sulfate proteoglycans, hyaluronic acid and other molecules, the levels of which are upregulated, affecting remyelination (Liddelow et al., 2017). On the other hand, a large number of astrocytes produce platelet-derived growth factor α and fibroblast growth factor 2, which inhibit the differentiation of OPCs. In this study, we found that the expression of GFAP was elevated in the MO group compared with the NC group (*p* < 0.001). At the three tested doses, of catalpol decreased GFAP expression in the brain to varying degrees (*p* < 0.01, Figure 7).

### Effects of Catalpol on NOTCH1 Signaling Pathway-Related Proteins and Genes in the Brains of Demyelinated Mice

The NOTCH1 signaling pathway is active in the nervous system throughout life and plays a role in maintaining the steady state of stem or progenitor cells in the developing CNS (Chitnis et al., 1995; Wettstein et al., 1997). NOTCH1 signaling alters the proliferation and differentiation of differentiated cells through cell-to-cell communication. The activation of NOTCH1 ligands and downstream target genes of the *Hes* family blocks the differentiation of OPCs, causing the failure of myelination by OLs. These findings indicate that the NOTCH1 pathway may be primarily responsible for the failure of remyelination in MS (Mathieu et al., 2019). Therefore, in this experiment, we mainly assessed the levels of several indicators related to the NOTCH1 signaling pathway: the ligand JAGGED1; the transmembrane receptor NOTCH1 (Fortini, 2009; Kovall et al., 2017); Notch intracellular domain (NICD), which is released by γ-secretase; recombination signal binding protein for immunoglobulin kappa J region (RBPJ), and the downstream transcription factors HES1 and HES5 (Tamura et al., 1995).

**FIGURE 8:**
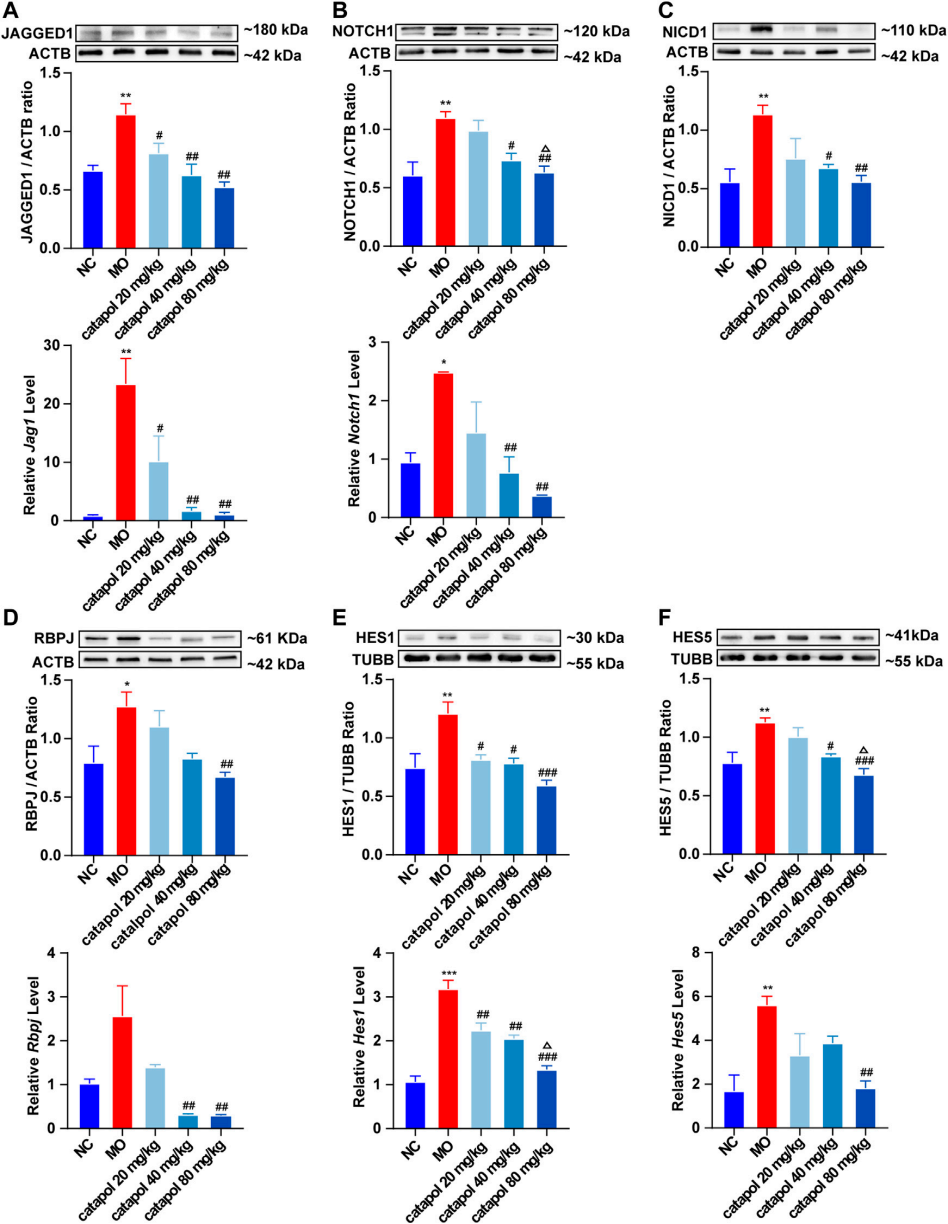
Catalpol at different concentrations down-regulated the expression of NOTCH1 signaling pathway-related proteins and genes in demyelinated mice. **(A)** The expression of JAGGED1 protein and *Jag1* gene in the brains, **(B)** the expression of NOTCH1 protein and *Notch1* gene in the brain, **(C)** the expression of NICD1 protein in the brain, **(D)** the expression of RBPJ protein and *Rbpj* gene in the brain, **(E)** the expression of HES1 protein and *Hes1* gene in the brain, **(F)** the expression of HES5 protein and *Hes5* gene. The relative expression of gene content was calculated by using 2^−△△Ct^ method. The data are expressed as mean ± SEM (n = 3/each group). Compared with NC group, *p < 0.05, **p < 0.01, ***p < 0.001; compared with MO group, ^#^ *p* < 0.05, ^##^ *p* < 0.01, ^###^ *p* < 0.001; compared with 20 mg/kg catalpol group, ^△^ *p* < 0.05.

We observed that the protein and gene levels of NOTCH1(*Notch1*), JAGGED1 (*Jag1*), NICD1, RBPJ (*Rbpj*), HES1(*Hes1*), and HES5 (*Hes5*) were significantly increased in mice in the MO group compared with mice in the NC group (*p* < 0.01). Since NICD1 is a digested fragment of the NOTCH1 protein, it cannot be regulated at the gene level. Thus, PCR analysis of this gene was not performed. However, after treatment with different concentrations of catalpol, the levels of the abovementioned markers decreased to varying degrees. The protein and gene expression of JAGGED1 (*Jag1*) was decreased in all three treatment groups compared to the MO group (*p* < 0.05, *p* < 0.001, Figure 8A); 40 mg/kg and 80 mg/kg catalpol reduced the expression of NOTCH1(*Notch1*), with 80 mg/kg catalpol having the strongest effect (*p* < 0.05, Figure 8B); the expression of NICD1 was lower in the brains of mice treated with 40 mg/kg or 80 mg/kg catalpol than those of mice in the MO group (*p* < 0.05, *p* < 0.01, Figure 8C); all doses of catalpol downregulated the expression of RBPJ (*Rbpj*) to a certain extent, with 80 mg/kg catalpol having the most significant effect (*p* < 0.05, Figure 8D); the protein and gene levels of HES1 (*Hes1*) in the brain were obviously decreased in the three catalpol treatment groups compared to the MO group, with 80 mg/kg catalpol having the most significant effect (*p* < 0.05, Figure 8E); and the downward trend in HES5 (*Hes5*) expression was similar to that of HES1 (*Hes1*) (Figure 8F).

### Cultivation of OPCs and OLs

**FIGURE 9:**
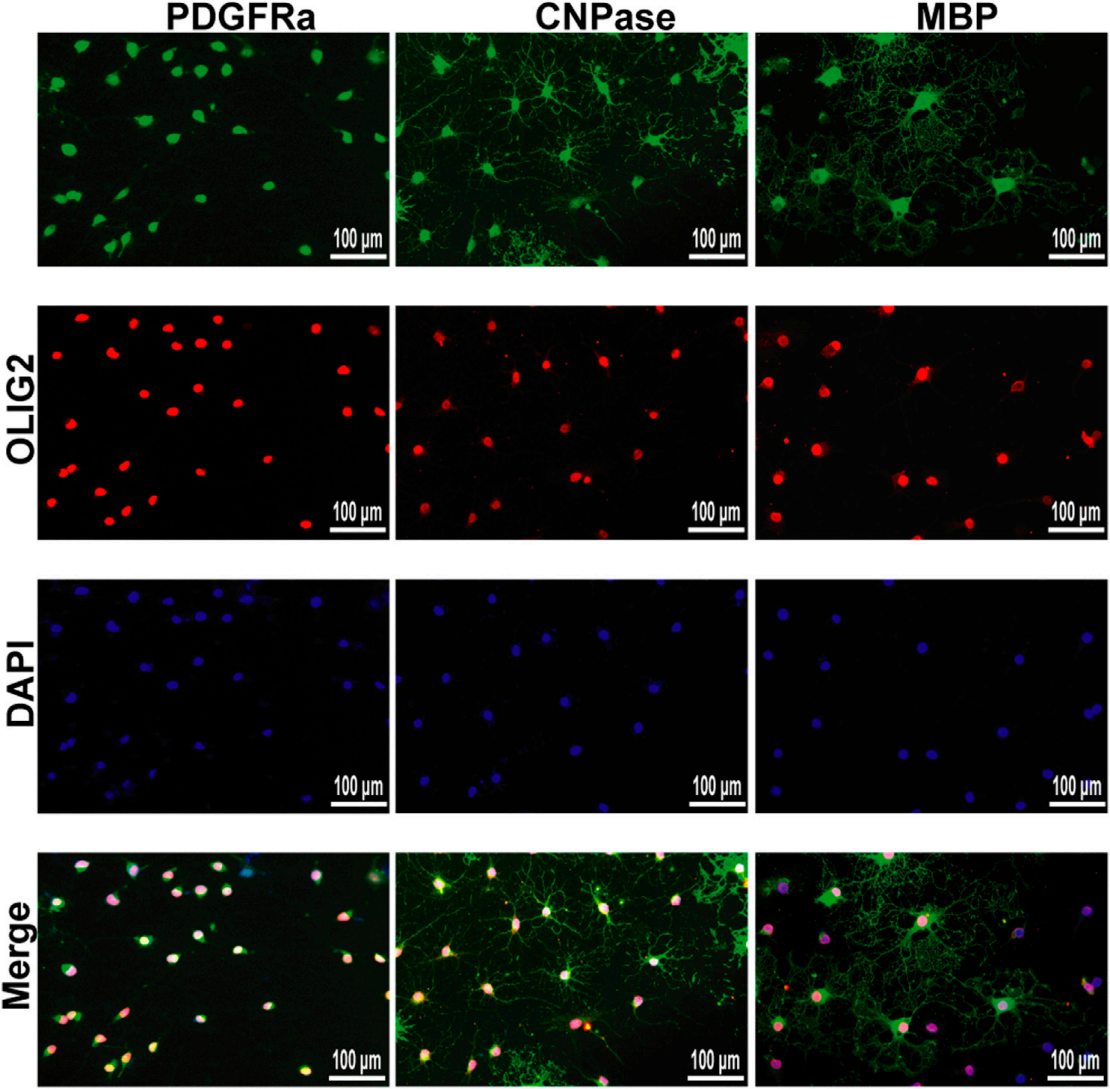
Immunofluorescence localization of PDGFRα, CNPase and MBP in the OPCs and OLs *in vitro*. PDGFRα, CNPase and MBP (green)were labeled with fluorescent secondary antibodies, and the nuclei were labeled with DAPI.

We successfully isolated OPCs from the brains of suckling rats and cultured them *in vitro*. We used PDGFRα, a marker of OPCs, to identify the cultured cells. Next, OPCs were cultured in differentiation medium, and the cells at different stages from OPCs to OLs were labeled with markers at different stages to prove that the cultured OPCs had strong differentiation ability (Figure 9).

### Catalpol Increased the Viability of Primary OPCs

**FIGURE 10:**
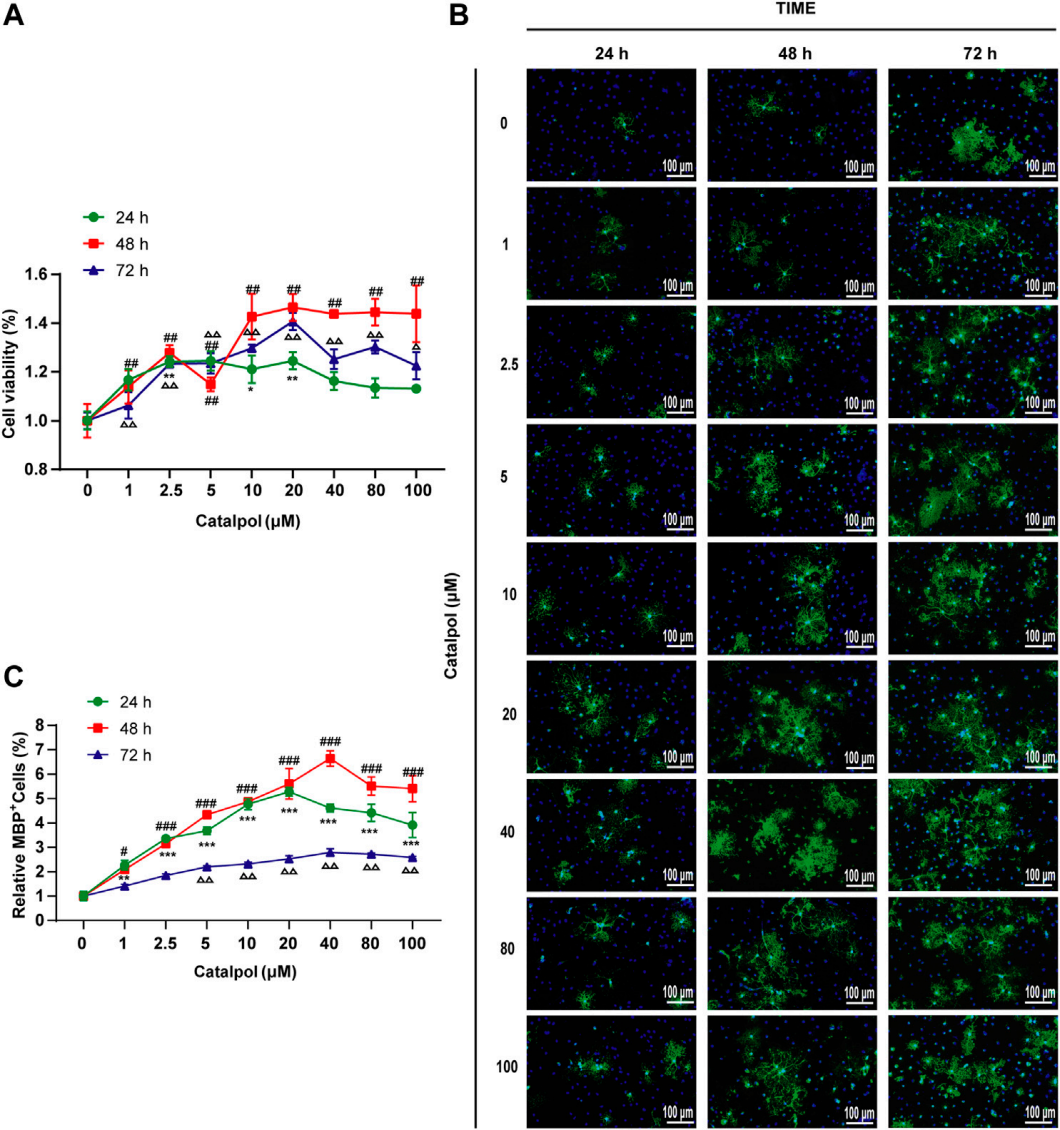
Catalpol promoted the proliferation and differentiation of OPCs *in vitro*. **(A)** OPCs were treated with catalpol (0, 1, 2.5, 5, 10, 20, 40, 80, 100 μM) for 24, 48 and 72 h. Cell viability was analyzed by CCK-8 assay. **(B)** Immunofluorescence localization of MBP. MBP (green) was labeled with fluorescent secondary antibodies, and the nuclei were labeled with DAPI. **(C)** The ratio of quantitative analysis of the fluorescent expression of MBP in each group to the untreated (Catalpol, 0 μM) group. The data are expressed as the mean ± SEM of three independent experiments, compared with catalpol 0 μM for 24 h group, *p < 0.05, **p < 0.01, ***p < 0.001; compared with catalpol 0 μM for 48 h group, ^#^ *p* < 0.05, ^##^ *p* < 0.01, ^###^ *p* < 0.001; compared with catalpol 0 μM for 72 h group, ^△^ *p* < 0.05, ^△△^ *p* < 0.001.

To study the effect of catalpol on the activity of OPCs, cells were treated with catalpol (1, 2.5, 5, 10, 20, 40, 80, or 100 μM) for 24, 48 or 72 , and cell viability was measured by the CCK-8 assay. Catalpol increased the viability of OPCs to different extents (Figure 10A).

### Catalpol Promoted the Formation of Mature OLs *In Vitro*

MBP is a marker of mature OLs. Catalpol significantly increased the number of MBP^+^ cells. The results revealed that treatment with 0–100 μM catalpol for 24–72 h promoted the differentiation of OPCs into OLs (Figure 10B).

According to the findings related to the viability of and MBP expression in OPCs, catalpol had the best effect at a concentration of 40 μM for 48 h (Figure 10C). Therefore, we chose treatment with 40 μM catalpol for 48 h for further experiments *in vitro*.

### Catalpol Promoted the Differentiation of OPCs *In Vitro* via the NOTCH1 Signaling Pathway

**FIGURE 11:**
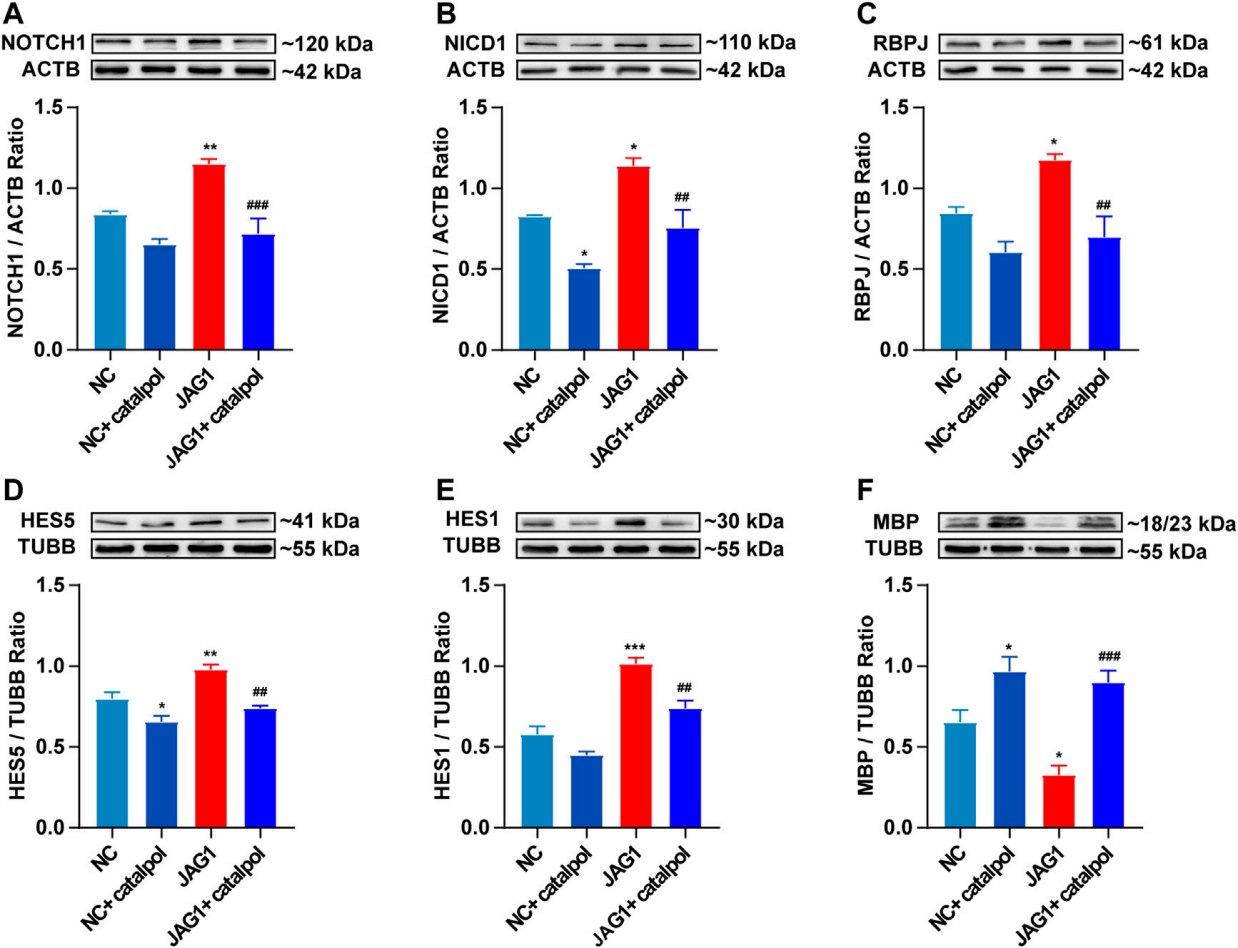
Catalpol promoted the differentiation of OPCs *in vitro* by inhibiting NOTCH1 signaling pathway **(A)** The expression of NOTCH1 protein in OPCs *in vitro*, **(B)** the expression of NICD1 protein in OPCs, **(C)** the expression of RBPJ protein in OPCs, **(D)** the expression of HES5 protein in OPCs, **(E)** the expression of HES1 protein in OPCs, **(F)** the expression of MBP protein in OPCs. The data are expressed as mean ± SEM of three independent experiments. Compared with NC group, *p < 0.05, **p < 0.01, ***p < 0.001; compared with JAG1 group, ^##^ *p* < 0.01, ^###^ *p* < 0.001.

To investigate whether the NOTCH1 signaling pathway can influence the differentiation of OPCs, OPCs were seeded in 6-well plates and treated with 40 μM catalpol in the absence or presence of the NOTCH1 signaling pathway agonist JAGGED1 polypeptide for 48 h. Treatment with 5 μM JAGGED1 for 24 h blocked the formation of MBP^+^ OLs, and the addition of catalpol reversed this effect (Figure 11F). The protein levels of NICD1, RBPJ, HES1 and HES5 effectively increased in OPCs in the JAGGED1 group (JAG1). The expression of the above indicators was significantly lower in cells treated with catalpol in the presence of JAGGED1 than in cells in JAG1 group (Figures 11A–E). Taken together, our findings indicate that the stimulation of OL formation by catalpol is in part due to the suppression of the NOTCH1 signaling pathway.

## Discussion

### Establishment of the CPZ-Induced Demyelination Model

CPZ is a copper ion-chelating agent that targets many metalloenzymes (such as ceruloplasmin), impairs the activity of copper-dependent cytochrome oxidase, and reduces oxidative phosphorylation, leading to degenerative changes in OLs (Cammer, 1999). These changes result in the apoptosis of mature OLS, causing extensive demyelination of the corpus callosum, internal capsule, thalamus and other white matter bundles (Blakemore, 1972; Clarner et al., 2015). However, after CPZ administration is stopped, the myelin protein is re-expressed. Therefore, although pathological changes in autoimmunity observed in MS can be stimulated by the EAE model, the demyelination model induced by the addition of CPZ to the diet, which also mimics the important histological features of demyelinating diseases (van der Star et al., 2012), is the ideal model for researching myelination in MS (Gudi et al., 2014). The exact dosage of CPZ has been determined by several studies. The most common protocol involves feeding 6-to 8-week-old mice with 0.2% CPZ for 5–6 weeks (Vega-Riquer et al., 2019). When 0.2%–0.6% CPZ is mixed with standard rodent food, a significant decrease in myelin protein is observed (Carlton, 1967). Studies have shown that increasing the dose of CPZ from 0.2% to 0.3% can significantly increase the degree of demyelination (Lindner et al., 2008) but increases the mortality rate of mice by more than 5%–10% (Torkildsen et al., 2008). Therefore, 0.2% CPZ is the most suitable concentration because it can cause extensive demyelination and fewer side effects (Liñares et al., 2006; Hesse et al., 2010; Skripuletz et al., 2011).

This experiment mainly studied the effect of catalpol on promoting myelination and neuroprotection, so the CPZ-induced demyelination model was selected. The results showed that after mice were fed a special diet containing 0.2% CPZ for 5 weeks, the mice exhibited a substantial decrease in weight, deficits in motor ability, and a reduction in LFB staining of myelin in the corpus callosum. The lamellar structure of the myelin sheath was obviously loose and separated, and the G-ratio value was increased. We found that after the demyelinated mice were fed a normal diet for 3 weeks beginning during the 6^th^ week, the weights and motor abilities of the mice slowly recovered, LFB staining of myelin in the corpus callosum increased, and the loosening of the myelin layers decreased, indicating that some myelin sheaths had regenerated.

### Neuroprotective Effects of Catalpol

When demyelinating injury occurs in MS, the damaged myelin fragments activate the immune inflammatory response and recruit a large amount of infiltrating immune inflammatory cells. Excessive inflammatory responses cause neuronal damage and severe neurological dysfunction in MS patients (Paganin and Ferrando, 2011). Catalpol exerts a strong neuroprotective effect and improves neurocognitive function (Xia et al., 2017) by reducing the level of proinflammatory cytokines and reducing oxidative stress in the nervous system, thereby slowing chronic inflammation and neurodegeneration (Wang et al., 2019). Previous studies have found that catalpol decreases immune inflammation and improves nerve damage in EAE mice (Yang et al., 2017). The results of this experiment revealed that at different doses, catalpol slowed the loss of myelin and the loosening of the myelin structure in the corpus callosum in mice, improved the body weight of the mice and increased the time spent on the rod by the mice in the rotarod test, showing that catalpol exerts good neuroprotective effects in mice with CPZ-induced demyelination. Catalpol had a better effect at 40 mg/kg and 80 mg/kg than at 20 mg/kg.

### Catalpol Promoted Remyelination

When demyelination occurs, axonal conduction is blocked (Waxman, 1977), and this effect is closely related to the functional defects observed after inflammatory demyelination in MS patients (Smith and McDonald, 1999; Jenkins et al., 2010). Therefore, prevention of demyelination and promotion of remyelination are the basic neuroprotective strategies for MS (Plemel et al., 2017; Villoslada and Steinman, 2020). In the mouse model of CPZ-induced demyelination, the regeneration of myelin in the injured area is closely related to functional recovery (Mozafari et al., 2010), which is extensive and rapid. The proliferation and recruitment of OPCs after injury to the mature CNS is very effective. Neural stem cells or progenitor cells from the subependymal zone rapidly produce OPCs that contribute to myelination (Xing et al., 2014), and OLs newly formed by OPCs undergo remyelination (Crawford et al., 2016). Studies have shown that the change between the average density of OPCs in chronically damaged sites in MS and normal sites is not obvious, which indicates that the number of OPCs may not be the limiting factor for remyelination in chronic injury, proving the importance of the differentiation of OPCs during remyelination (Hughes et al., 2013).

During the process of myelination in MS, OLIG2 acts as a cell transcription factor throughout the differentiation of OL lineage cells and can simultaneously label OPCs and OLs. The experimental results showed that the expression of OLIG2 in the corpus callosum increased slightly in all demyelinated mice, but there was no significant difference between the groups, indicating that OPCs in the demyelinated area were not lacking. CNPase is expressed in the middle stage of differentiation, while MBP is a marker of mature OLs. Early *in vitro* studies have found that catalpol increases the expression of OLIG1 in isolated OPCs and promotes the differentiation and maturation of OPCs (Yuan et al., 2015). This experiment revealed that catalpol increased the number of CNPase^+^/OLIG2^+^ and MBP^+^/OLIG2^+^ cells in the demyelination site and that 40 μM catalpol promoted the differentiation of OPCs *in vitro*. The results provide an objective basis for catalpol to promote myelination.

### Catalpol Promoted Remyelination by Regulating the NOTCH1 Signaling Pathway

In MS, remyelination is always insufficient. The failure of myelination appears to be the result of a variety of pathological processes that interfere with the maturation of OPCs. The activation of the NOTCH1 signaling pathway is one of these processes (Mathieu et al., 2019). It has been reported that the NOTCH1 signaling axis is activated in response to TGF-β in the brains of MS patients and in cocultured astrocytes and OPCs *in vitro*. As in normal development, JAGGED1 is expressed in astrocytes and neurons in chronic demyelinating lesions, and the receptor NOTCH1 on OPCs combines with activated JAGGED1 and undergoes a conformational change. Subsequently, ADAM metalloprotease mediates the first protein cleavage, producing an intermediate protein hydrolysate called Notch EXtracellular Truncation (NEXT). NEXT is the substrate for the γ-secretase complex, which releases NICD. NICD passes through the cytoplasm from the inner membrane of the plasma membrane to the nucleus. In the cell nucleus, after NICD combines with RBPJ, RBPJ is converted into a transcription activator that recruits acetyltransferase p300 and activates the downstream transcription factors HES1 and HES5 (Tamura et al., 1995). The transcription factors HES1 and HES5 inhibit the maturation of OPCs and maintain their differentiation status. Therefore, blocking NOTCH1 signaling may enhance remyelination.

In EAE mice, the γ-secretase inhibitor MW167 can effectively inhibit the NOTCH1 pathway and promote remyelination (Jurynczyk et al., 2008). In mice with focal demyelination caused by injection of lysophosphatidylcholine, specific knockout of *NOTCH1* in OPCs can significantly improve myelin repair (Zhang et al., 2009). *NOTCH1* siRNA can obviously promote OPC differentiation and promote remyelination to improve the symptoms of nerve injury in CPZ mice (Fan et al., 2018). The above studies revealed that inhibiting the NOTCH1 signaling pathway can improve the disease pathology of MS in animal models and enhance the regeneration of the myelin sheath. Other studies have found that when the expression of jagged1 in astrocytes is induced by TGFbeta1, OL differentiation increases (Zhang et al., 2010). Adding 10 µM DAPT (a γ-secretase inhibitor) to an OPC and astrocyte coculture system for 6 h can significantly inhibit the expression of NICD and promote cell differentiation (Wang et al., 2017). This study showed that *in vivo*, catalpol significantly promoted remyelination in CPZ-treated mice and decreased the expression of the NOTCH1 signaling pathway proteins JAGGED1, NOTCH1, NICD1, and RBPJ and the downstream target genes *Hes1* and *Hes5* and 80 mg/kg catalpol had the best therapeutic effect. Multiple studies have proven that inhibition of the NOTCH1 signaling pathway can promote the differentiation and maturation of OPCs *in vivo* and *in vitro* and promote the remyelination of demyelinated mice. Furthermore, this study revealed that the NOTCH1 pathway is strongly activated in OPCs upon treatment with 5 μM JAGGED1 polypeptide for 24 h. The number of MBP^+^ cells as also significantly reduced. Treatment with 40 μM catalpol for 48 h greatly reversed this effect. Catalpol (40 μM) downregulated the expression of NOTCH1 pathway-related proteins, such as NOTCH1, NICD1, RBPJ, HES1, and HES5, and promoted the differentiation of OPCs. Therefore, our results clearly indicated that the effect of catalpol in promoting remyelination may be related to downregulation of the expression of NOTCH1 signaling pathway-related indicators.

In conclusion, catalpol exerts obvious neuroprotective effects in mice with CPZ-induced demyelination, promoting remyelination. Its mechanism may involve regulation of the NOTCH1 signaling pathway. However, further in-depth exploration of the mechanism is required.

## Data Availability

The original contributions presented in the study are included in the article/Supplementary Material, further inquiries can be directed to the corresponding author.
